# General Population Awareness of Primary Immune Deficiency Disease in Children in the Arar Region, Saudi Arabia

**DOI:** 10.7759/cureus.54102

**Published:** 2024-02-13

**Authors:** Safya E Esmaeel, Hassan T Mohamed, Reef A Alshammari, Israa S Alanazi, Naseem D Alrawaili, Fai S Alanazi

**Affiliations:** 1 Physiology, Northern Border University, Arar, SAU; 2 Pediatrics, Maternity and Children Hospital, Arar, SAU; 3 Medicine, Northern Border University, Arar, SAU

**Keywords:** arar city, awareness, children, pidd, immune deficiency

## Abstract

Background

Primary immunodeficiency disorders (PIDD) are of various types and severities, and they are associated with a delay in diagnosis. Early diagnosis of PIDD helps to improve the quality of life of affected children and prevent permanent consequences such as organ damage. Hence, awareness of PIDD is a must in the community to aid in early detection.

Objectives

The study aims to investigate the general population's awareness of PIDD in children in Arar, Northern Saudi Arabia.

Methods

A cross-sectional design was utilized to determine the awareness of PIDD in children in Arar, Northern Saudi Arabia. The participants were selected through an online self-administered questionnaire. The collected data was analyzed using descriptive and inferential statistics.

Results

A total of 528 participants were involved in the current study. The majority of the sample population falls within the 20-30 age range. 9.1% of respondents know a child with primary immunodeficiency. Additionally, participants were aware of certain symptoms, such as delayed growth and chronic diarrhea, with rates of 47.0% and 34.1%, respectively. On the other hand, symptoms like otitis media and sinusitis have lower awareness rates of 25.8% and 33.3%, respectively.

Conclusion

This study can help in developing targeted awareness campaigns and educational programs to improve the understanding of primary immune deficiency disease among the general population in Saudi Arabia. This, in turn, can lead to earlier diagnosis and better management of the disease in children, ultimately improving their quality of life.

## Introduction

Primary immunodeficiency diseases (PIDD) comprise a group of more than 300 distinct diseases arising from different genetic abnormalities that affect the development and/or function of the immune system [1]. The exact prevalence of PIDD is uncertain because of the lack of screening, national registries, or reporting by government health surveys in many countries; however, a PIDD prevalence of approximately 1:10.000 was reported in Australian, North American, and European populations [2].

Primary immunodeficiency diseases are genetic disorders that predispose patients to frequent infections, autoimmunity, and malignancies; they are considered to be rare disorders worldwide. Therefore, establishing a database is important to determine the magnitude, types, and spectrum of PIDD disease encountered in a certain population. Similar databases worldwide have shown geographical and racial variation in the spectrum of PIDDs [3, 4]. Registry data from Saudi Arabia are limited to two studies. Both are from a homogeneous population and from only one region of the country and, hence, likely do not represent the whole nation [5].

Nevertheless, the elevated rate of consanguineous marriages was shown in the studies of a relatively large number of patients diagnosed with an autosomal recessive inherited form of PIDDs as in severe combined immunodeficiency (SCID), hyper-IgE syndrome, and hyper-IgM syndrome [6]. Therefore, Saudi population data is important in particular to autosomal recessive inherited PIDDs [7].

Moreover, the overall incidence of combined immunodeficiency (CID) is estimated to be 1 in 75,000 to 100,000 live births. Since most forms of CID are inherited as autosomal recessive traits, anecdotal experience suggests that the incidence of CID is higher in our region than in Western countries, probably exceeding 1 in 10,000 live births [8,9]. However, accurate epidemiologic data remains scarce.

There are important preliminary results, and they highlight the need for ongoing, systematic data collection to learn more about PIDDs in Saudi Arabia. Furthermore, consanguineous marriages in Saudi Arabia are high. This has provided a background for genetic diseases to be abundant in the Saudi population. Hence, awareness of primary immune deficiency (PIDD) in children in Saudi Arabia is necessary. The study aims to investigate the Saudi general population's awareness of primary immune deficiency disease in children in Arar, Northern Saudi Arabia.

## Materials and methods

Study design, setting, and duration

This study is a community-based cross-sectional study was conducted in Arar, Saudi Arabia, in October 2023.

Target population, sampling, and duration of study

The adult general population above 18 years in the Northern Border Region. The sample size is calculated using formula n= (z^2 x pq)/d^2, where n represents the sample size, z is the standard deviation (1.96), p is the prevalence (0.5), q is 1-p, DE is the design effect (2), and d is the accepted error (0.05), was determined 520. Participants were selected through non-probability convenience sampling. The study lasted for four months.

Data collection Tool

An online self-administered questionnaire including 5 sections (demographic data, awareness of risk factors, awareness of manifestation, awareness of complications and awareness of diagnosis and treatment of PIDD prepared in Arabic after reading and accepting the informed consent and distributed via an anonymous online survey instrument, which will target population who live in Arar, Saudi Arabia [10].

There was a pilot study for the questionnaire to finalize it to assess the general awareness of PIDD in children.

Inclusion and exclusion criteria

The study included Saudi and non-Saudi nationals aged ≥18 years, mentally competent, and residing in Arar, in the Northern Border Region. Exclusions were made for those below 18 years of age or who are unable to respond to the questionnaire.

Sample size and data analysis

We estimated a sample size of 520 participants using the Raosoft® calculator (Raosoft, Inc., Seattle, Washington), with a 5% level of significance, 5% margin of error, 95% confidence, and expected response distribution of 50%. Data analysis was done using SPSS (IBM Inc., Armonk, New York).

Pilot Study

A pilot study was conducted on 10% of the gathered sample to test the reliability and applicability of the study to ascertain the feasibility, applicability, and clarity of the tool, and no modifications were made. Participants in the pilot study were excluded from the study.

Statistical analysis

Data were analyzed using SPSS version 26. Association was tested with the Chi-square test. Qualitative variables were represented as percentages and numbers (mean, frequency, etc.) and shown in the figures. A 0.05 level of significance was used in all tests used in the study.

## Results

Table 1 shows that the majority of the sample population falls within the 20-30 age range, accounting for 31.4% of the total. This is followed by the 31-40 and 41-50 age groups, each comprising 18.9% and 18.6% of the population, respectively. Notably, individuals under the age of 20 make up 12.9% of the sample, while those aged 51-60 constitute 18.2% of the total. In terms of gender distribution, females represent a significant majority at 72.0% compared to males at 28.0%. The nationality breakdown shows that the overwhelming majority of the sample population is Saudi, accounting for 95.1% of the total. Non-Saudi individuals make up the remaining 4.9%. The educational level distribution reveals that a significant proportion of the sample population has attained a university degree or higher, constituting 87.9% of the total. This is followed by high school graduates at 8.0%, middle school graduates at 2.7%, and individuals with elementary school or uneducated backgrounds, each representing less than 1% of the population. Moving on to marital status, the data indicates that the majority of the sample population is married, accounting for 61.4% of the total. Single individuals represent 35.6%, while divorced and widowed individuals make up 1.9% and 1.1%, respectively. The parental status breakdown shows that 59.8% of the sample population have children, while 40.2% do not. Furthermore, the distribution of the number of children among those who are parents provides valuable insights into family size and dynamics within the population.

**Table 1:** Sociodemographic characteristics of participants (n=528)

Parameter		n	%
Age	less than 20	68	12.9
	20-30	166	31.4
	31-40	100	18.9
	41-50	98	18.6
	51-60	96	18.2
Gender	Male	148	28.0
	Female	380	72.0
Nationality	Saudi	502	95.1
	Non-Saudi	26	4.9
Education level	Uneducated	4	.8
	Elementary school	4	.8
	Middle school	14	2.7
	High school	42	8.0
	University or higher	464	87.9
Marital status	Married	324	61.4
	Single	188	35.6
	Divorced	10	1.9
	Widowed	6	1.1
Have children	Yes	316	59.8
	No	212	40.2
Number of children	1.0	30	5.7
	2.0	40	7.6
	3.0	38	7.2
	4.0	46	8.7
	5.0	58	11.0
	6.0	50	9.5
	7.0	34	6.4
	8.0	10	1.9
	9.0	2	.4
	10.0	2	.4
	11.0	4	.8
	12.0	2	.4

Table 2 shows that a proportion of respondents (9.1%) know a child with primary immunodeficiency. Furthermore, the data indicates that 15.5% of children have taken medications previously. The completion rate of vaccination doses among children is notably high at 69.7%, but it also indicates that over 30% have not completed their vaccination. Also, The presence of abnormal signs during clinical examinations in 2.7% of cases. Additionally, it shows the prevalence of a family history of primary immunodeficiency (3.8%) and recurrent infections (10.6%).

**Table 2:** Prevalence, family history, and risk factors of PIDD among participants' children (n=528) PIDD - primary immunodeficiency disorders

	Yes, n (%)	No, n (%)
Have a child with primary immunodeficiency	0 (0%)	528 (100.0%)
Know a child with primary immunodeficiency	48 (9.1%)	480 (90.9%)
Family history of primary immunodeficiency	20 (3.8%)	508 (96.2%)
Family history of recurrent infections	56 (10.6%)	472 (89.4%)
Your child taken medications before	82 (15.5%)	446 (84.5%)
Your child completed their vaccination doses	368 (69.7%)	160 (30.3%)
Abnormal signs obtained during the clinical examination of child	14 (2.7%)	514 (97.3%)

In Table 3, percentages associated with each symptom awareness indicate the frequency with which they occur in children with PIDD. It is evident that participants were aware of certain symptoms, such as delayed growth and chronic diarrhea, with rates of 47.0% and 34.1%, respectively. On the other hand, symptoms like otitis media and sinusitis have lower awareness rates of 25.8% and 33.3%, respectively.

**Table 3:** Knowledge of participants of symptoms of PIDD in children (n=528) PIDD - primary immunodeficiency disorders

	Yes, n (%)	No, n (%)
Otitis media is a symptom of PIDD in children	136 (25.8%)	392 (74.2%)
Sinusitis is a symptom of PIDD in children	176 (33.3%)	352 (66.7%)
Pneumonia is a symptom of PIDD in children	220 (41.7%)	308 (58.3%)
Asthma is a symptom of PIDD in children	238 (45.1%)	290 (54.9%)
Bronchiectasis is a symptom of PIDD in children	172 (32.6%)	356 (67.4%)
Delayed growth is a symptom of primary immunodeficiency PIDD in children	248 (47.0%)	280 (53.0%)
Chronic diarrhea is a symptom of PIDD in children	180 (34.1%)	348 (65.9%)
Septicemia is a symptom of primary immunodeficiency PIDD in children	236 (44.7%)	292 (55.3%)
Meningitis is a symptom of primary immunodeficiency syndrome in children	238 (45.1%)	290 (54.9%)
Abnormal gait is a symptom of PIDD in children	174 (33.0%)	354 (67.0%)
Arthritis is a symptom of PIDD in children	184 (34.8%)	344 (65.2%)
Dermatitis is a symptom of primary immunodeficiency in children	204 (38.6%)	324 (61.4%)
Eczema is a symptom of primary immunodeficiency in children	200 (37.9%)	328 (62.1%)
Superficial abscess is a symptom of PIDD in children	152 (28.8%)	376 (71.2%)
Deep abscess is a symptom of primary immunodeficiency syndrome in children	174 (33.0%)	354 (67.0%)
Fungal infections of the mouth are a symptom of PIDD in children	210 (39.8%)	318 (60.2%)
Abnormal tooth growth is a symptom of PIDD in children	164 (31.1%)	364 (68.9%)
Liver enlargement is a symptom of PIDD in children	206 (39.0%)	322 (61.0%)
Spleen enlargement is a symptom of PIDD in children	204 (38.6%)	324 (61.4%)
Lymphadenitis is a symptom of PIDD in children	200 (37.9%)	328 (62.2%)
Severe infection from a vaccination is a symptom of PIDD in children	192 (36.4%)	336 (63.6%)

According to Table 4, the respondents believe that delayed growth, failure of body systems, and death from a serious infection are complications of PIDD in children, with percentages ranging from 43.9% to 47.3% for "Yes" responses. On the other hand, the respondents believe that autoimmune disorders, cancer, and death from a serious infection are linked to PIDD in children, with percentages ranging from 45.5% to 47.0% for "Yes" responses.

**Table 4:** Knowledge of participants of complications of PIDD in children (n=528) PIDD - primary immunodeficiency disorders

	Yes, n (%)	No, n (%)
Autoimmune disorder is a complication of PIDD in children	246 (46.6%)	282 (53.4%)
Delayed growth is a complication of PIDD in children	232 (43.9%)	296 (56.1%)
Cancer is a complication of PIDD in children	240 (45.5%)	288 (54.5%)
Failure of body system is a complication of PIDD in children	250 (47.3%)	278 (52.7%)
Death from a serious infection is a complication of PIDD in children	248 (47.0%)	280 (53.0%)

Table 5 indicates that a significant majority, 66.3%, believe that blood tests are important for diagnosing PIDD in children, while 49.6% consider prenatal testing to be important for the same purpose. Moving on to treatment methods, the data reveals an almost evenly split opinion on the efficacy of stem cell transplantation for PIDD in children, with 50.4% in favor and 49.6% against. Similarly, there is a clear divide regarding the use of gene therapy as a treatment method, with 56.4% expressing belief in its effectiveness and 43.6% expressing skepticism. The final statement addresses premarital screening as a preventive measure for PIDD in children. The data shows that a substantial majority, 63.6%, view premarital screening as a crucial preventive measure for PIDD in children.

**Table 5:** Knowledge of participants of diagnosis, management, and prevention of PIDD in children (n=528) PIDD - primary immunodeficiency disorders

	Yes, n (%)	No, n (%)
Blood test is important for diagnosing PIDD in children	350 (66.3%)	178 (33.7%)
Prenatal testing is important for diagnosing PIDD in children	262 (49.6%)	266 (50.4%)
Stem cell transplantation is a good treatment for PIDD in children	266 (50.4%)	262 (49.6%)
Gene therapy is a treatment method for PIDD in children	230 (43.6%)	298 (56.4%)
Premarital screening is a preventive measure for PIDD in children	336 (63.6%)	192 (36.4%)

As illustrated in Table 6, it is evident that there is a statistically significant association between age and the uptake of premarital screening, with a p-value of 0.001. The percentage of individuals undergoing premarital screening appears to decrease as age increases. For example, in the age group less than 20, 68 individuals participated in premarital screening, representing 12.9% of the total sample in this age group. In contrast, in the age group 51-60, only 46 individuals (8.7%) participated in premarital screening. When considering marital status, there seems to be no statistically significant association between marital status and premarital screening uptake, as indicated by the p-value of 0.180. However, it is worth noting that the percentage of married individuals undergoing premarital screening is relatively higher compared to other marital status categories. Gender also appears to be significantly associated with premarital screening uptake, with a p-value of 0.001. The data suggests that a higher percentage of females (51.9%) have undergone premarital screening compared to males (11.7%). In terms of nationality, there is no statistically significant association between nationality and premarital screening uptake, as indicated by the p-value of 0.820. However, it is important to note that a much higher percentage of Saudi nationals (60.6%) have undergone premarital screening compared to non-Saudi individuals (3.0%). Education level also shows a statistically significant association with premarital screening uptake, with a p-value of 0.015. The data indicates that a higher percentage of individuals with a university education or higher (54.2%) have undergone premarital screening compared to those with lower levels of education. Lastly, the presence of children does not appear to have a statistically significant association with premarital screening uptake, as indicated by the p-value of 0.365. The percentage of individuals with children undergoing premarital screening is similar to those without children.

**Table 6:** Knowledge of participants of PIDD screening in association with their sociodemographic characteristics (n=528) PIDD - primary immunodeficiency disorders

Characteristics		Premarital screening is a preventive measure for PIDD in children		Total (N=528)	p-value
		Yes, n (%)	No, n (%)		
Age	Less than 20	46 (8.7%)	22 (4.2%)	68 (12.9%)	0.001
	20-30	98 (18.6%)	68 (12.9%)	166 (31.4%)	
	31-40	76 (14.4%)	24 (4.5%)	100 (18.9%)	
	41-50	70 (13.3%)	28 (5.3%)	98 (18.6%)	
	51-60	46 (8.7%)	50 (9.5%)	96 (18.2%)	
Marital status	Single	116 (22.0%)	72 (13.6%)	188 (35.6%)	0.18
	Married	206 (39.0%)	118 (22.3%)	324 (61.4%)	
	Divorced	8 (1.5%)	2 (0.4%)	10 (1.9%)	
	Widowed	6 (1.1%)	0 (0.0%)	6 (1.1%)	
Gender	Male	62 (11.7%)	86 (16.3%)	148 (28.0%)	0.001
	Female	274 (51.9%)	106 (20.1%)	380 (72.0%)	
Nationality	Saudi	320 (60.6%)	182 (34.5%)	502 (95.1%)	0.82
	Non-Saudi	16 (3.0%)	10 (1.9%)	26 (4.9%)	
Education level	Uneducated	2 (0.4%)	2 (0.4%)	4 (0.8%)	0.015
	Elementary school	2 (0.4%)	2 (0.4%)	4 (0.8%)	
	Middle school	14 (2.7%)	0 (0.0%)	14 (2.7%)	
	High school	32 (6.1%)	10 (1.9%)	42 (8.0%)	
	University or higher	286 (54.2%)	178 (33.7%)	464 (87.9%)	
Have children	Yes	206 (39.0%)	110 (20.8%)	316 (59.8%)	0.365
	No	130 (24.6%)	82 (15.5%)	212 (40.2%)	

## Discussion

It is important to raise awareness about primary immunodeficiency disorders in children in Saudi Arabia. PIDD is a group of more than 400 rare, chronic disorders in which part of the body's immune system is missing or functions improperly. This can lead to increased susceptibility to infections, autoimmune diseases, and other health complications.

According to our study, there is a lack of general population awareness about PIDD in children. This lack of awareness can lead to delays in diagnosis and treatment, which can have serious consequences for affected children. In a prior study, they discovered that there was a lack of understanding of the unique indications of PIDDs, namely the confirmation of Nijmegen breakage syndrome, ataxia-telangiectasia, and Di George syndrome [11]. The frequency of right answers about PIDD warning indicators in children increased significantly (85.1% versus 64.6% in 2016, P0.0001), while there was no significant gain in adult awareness about warning signs [12]. This is congruent with a Brazilian study, which also found a lack of awareness of primary immunodeficiency warning symptoms [13].

Moreover, the presence of abnormal signs during clinical examinations in 2.7% of cases underscores the importance of regular medical assessments for early detection and intervention, which is similar to our study [14].

In our study, 66.3% believe that blood tests are important for diagnosing PIDD in children, while 49.6% consider prenatal testing to be important for the same purpose. Moving on to treatment methods, the data reveals an almost evenly split opinion on the efficacy of stem cell transplantation for PIDD in children, with 50.4% in favor and 49.6% against. In a prior study, 44.6% of physicians reported ordering ancillary testing when dealing with PIDD patients, with laboratory tests such as serum immunoglobulin levels and differential lymphocyte count being the most common [15]. In another study, the most commonly reported tests were complete blood count and serum immunoglobulin levels (96%), followed by IgG subclass levels and chest X-ray (76.5%) [16, 17]. Even in industrialized countries, the diagnosis is frequently made after several years of symptoms, when the patient is hospitalized, or when the patient already has sequelae from the infections. To help non-immunologist physicians diagnose and initiate investigations, detailed protocols with clinical and laboratory information have been provided [12].

In addition, the varying percentages of individuals with different numbers of children, ranging from 5.7% for those with one child to 0.4% for those with 10 or more children, highlight the diversity in family sizes and can be informative for studies related to fertility, family planning, and child-rearing practices [18].

It is crucial for parents, caregivers, and healthcare professionals to be aware of the signs and symptoms of PIDD so that affected children can receive timely and appropriate medical care. Some common signs and symptoms of PIDD in children include frequent and/or severe infections, infections that are difficult to treat, infections that recur or do not fully clear up, and infections that affect multiple organs. Other signs may include failure to thrive, poor growth, and autoimmune conditions such as eczema, asthma, and allergies. It is important for parents and caregivers to be vigilant and seek medical attention if they notice any of these symptoms in their children.

In addition to raising awareness among parents and caregivers, it is also important to educate healthcare professionals about PIDD in children. This can help ensure that affected children are properly diagnosed and treated. Healthcare professionals should be aware of the different types of PIDD, their symptoms, and the appropriate diagnostic tests and treatments.

Furthermore, increasing awareness about PIDD in children can also help reduce the stigma and misconceptions surrounding the disease. Many people may not be familiar with PIDD and may have misconceptions about its causes and implications. By raising awareness and providing accurate information, we can help dispel these misconceptions and provide support to affected children and their families.

## Conclusions

There is a common lack of understanding regarding PIDD in children. This lack of understanding can cause delays in diagnosis and treatment, which can have major effects on the children who are affected. Raising PIDD awareness in the population is critical for assuring timely diagnosis and treatment, decreasing stigma, and giving support to affected children and their families in Saudi Arabia. We can assist improve the lives of children with PIDD and ensure that they receive the care and support they require by educating the general public, parents, and healthcare professionals.
